# Software-Defined Optical Coherence Measurement of Seawater Refractive Index Variations

**DOI:** 10.3390/s25103119

**Published:** 2025-05-15

**Authors:** Jiaxin Zhao, Xinyi Zhang, Qi Wang, Liyan Li, Songtao Fan, Yongjie Wang, Yan Zhou

**Affiliations:** 1 Optoelectronic Systems Laboratory, Institute of Semiconductors, Chinese Academy of Sciences, Beijing 100083, China; zhaojiaxin22@semi.ac.cn (J.Z.); zhangxinyi@semi.ac.cn (X.Z.); wq13301074549@semi.ac.cn (Q.W.); fstao@semi.ac.cn (S.F.);; 2 School of Electronic, Electrical and Communication Engineering, University of Chinese Academy of Sciences, Beijing 100049, China; 3 College of Materials Science and Optoelectronics Technology, University of Chinese Academy of Sciences, Beijing 100049, China

**Keywords:** software-defined radio, refractive index change, signal demodulation, interferometry

## Abstract

The seawater refractive index is an important parameter in marine environments, with its variations depending on the specific environmental conditions. During practical applications, modulation parameters such as the sampling rate, bandwidth, and filters directly affect the signal-to-noise ratio (SNR) and need to be adjusted in real-time according to the characteristics of the target signal. Low-cost software-defined radio (SDR) offers significant advantages in this regard. This paper proposes an optical coherence measurement method for seawater refractive index changes based on orthogonal demodulation using SDR along with simulation calculations, and the results demonstrate that the resolution of the refractive index change rate is $3.165\times 10^{-9}$ RIU/s, corresponding to a refractive index change resolution of $10^{-10}$ RIU (frequency range 1 Hz–100 Hz, measurement range 0.1 m). By adopting SDR as the implementation platform for the demodulation algorithm and using a radio-frequency source to simulate interference signals for demodulating the refractive index variation, the results show that the relative error of the SDR demodulation results is below 0.3%. Additionally, this study developed a software-defined optical coherence measurement system for the seawater refractive index and measured the refractive index changes in deionized water during heating. The experimental results showed that the root mean square error (RMSE) of the refractive index changes obtained through SDR demodulation was $5.68\times 10^{-6}$ RIU. This research provides a novel demodulation method for high-precision measurements of seawater refractive index changes under different marine environments.

## 1. Introduction

The refractive index of seawater, which reflects key parameters such as temperature, salinity, and density, is of significant importance in marine environmental monitoring. High-precision measurements of seawater refractive index have become a critical challenge in marine environmental monitoring techniques [1,2,3,4]. According to studies by Rusby and Stanley on the same seawater [5,6], when the temperature remains constant, a change of 1‰ in salinity results in a refractive index variation of $2\times 10^{-4}$ RIU. In the North Atlantic, during winter, a 1 °C change in seawater temperature causes a refractive index change of $5\times 10^{-5}$ RIU; during summer, a 1 °C temperature change results in a refractive index variation of $1\times 10^{-4}$ RIU. Refractive index variations also differ across different marine regions at the same time due to factors such as ocean currents and solar radiation [7]. According to the actual measurement data of Japanese scientists, in the deep-sea area along 149° E in the western North Pacific, the meridional gradient of the refractive index is that it changes by $4\times 10^{-8}$ RIU per 100 km in the area from (149° E, 23° N) to (149° E, 26° N). The zonal gradient is that the change in the seawater refractive index is $2.1\times 10^{-7}$ RIU per 100 km [8]. The measurement accuracies that the existing optical measurement methods for refractive index changes can achieve, such as the laser deflection method [9], the surface plasmon resonance method [10], the fiber Bragg grating method [11], and the critical angle method [12], are $1.5\times 10^{-6}$, $5\times 10^{-5}$, $5\times 10^{-3}$ RIU, and $1.2\times 10^{-5}$ RIU, respectively. The method based on laser interferometry can reach a maximum accuracy of $10^{-8}$ RIU, which is the highest known accuracy at present [13,14].

Signal demodulation is a crucial part of interferometric measurement, and its demodulation accuracy directly affects the measurement accuracy of the final refractive index change. During the signal processing, parameters such as the sampling rate, bandwidth, and filters, as well as the demodulation method, directly influence the signal-to-noise ratio of the demodulation [15,16]. We need to adjust the demodulation algorithm and the parameters in the demodulation measurement process according to different marine environments, different target signals, and hardware configurations. The software-defined radio (SDR) platform was first applied in the field of radio communication. After the signal is down-converted to a low-intermediate frequency signal through hardware, its main demodulation functions are implemented digitally and controlled by software. Therefore, it can flexibly and quickly adapt to various demodulation schemes, realizing the flexible configuration of demodulation parameters such as bandwidth and sampling rate, and showing strong advantages in demodulation adaptability [17]. In 2017, Riobó et al. first demonstrated the feasibility of using SDR for optical coherent signal demodulation [18]. They built a Michelson interferometer and measured the displacement between 3.5 pm and 122 pm, with a relative measurement uncertainty of 7% [19]. In 2019, Insabella et al. proposed software-defined optoelectronic two-dimensional photoacoustic tomography, with a noise-equivalent power (NEP) density as low as 1.41 mPa/Hz^1^/^2^ [20]. In 2023, Ce Zhang et al. realized the measurement of graphene nanoscale vibration based on SDR [21]. Although the optical coherent measurement technology based on software-defined radio (SDR) emerged later than traditional measurement technologies, it has broad development prospects due to its high flexibility.

To adapt to diverse marine environments, this paper proposes a digital quadrature demodulation algorithm based on software-defined radio (SDR). It theoretically analyzes the SDR-based demodulation algorithm and conducts simulations. By using a radio-frequency source to simulate the interference signal to demodulate the target refractive index change, an effect is achieved where the relative error of electrical demodulation is only 0.3% when the refractive index change is in the range from $10^{-5}$ RIU to $10^{-8}$ RIU. The corresponding demodulation accuracy is higher than that of the laser deflection method, the surface plasmon resonance method, the fiber Bragg grating method, and the critical angle method. Moreover, a system for measuring the refractive index change based on the laser interference method is built, and SDR is used for digital quadrature demodulation. Taking deionized water during the heating process as the test target, the experimental results show that the root mean square error (RMSE) of the refractive index change obtained by SDR demodulation is $5.68\times 10^{-6}$ RIU.

## 2. Principle of Seawater Refractive Index Variation Measurement Based on SDR

### 2.1. Optical System for Laser Heterodyne Coherent Refractive Index Measurement

**Figure 1:**
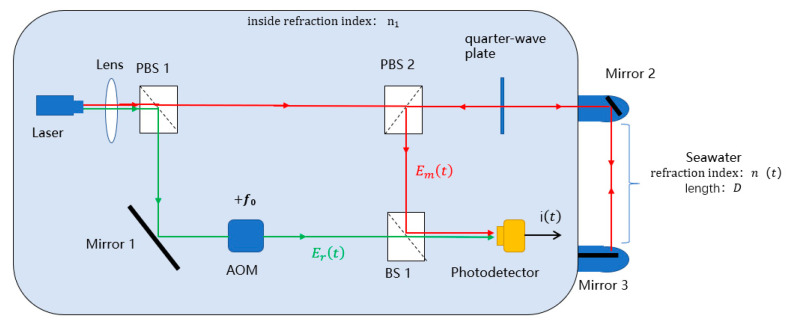
Schematic of the heterodyne interferometric system for refractive index measurement. The measurement beam is represented by red arrows, and this beam passes through the seawater measurement area. The reference beam is represented by green arrows. The black arrow represent the measurement signals output from the interferometric system.

Interferometric methods for measuring refractive index mainly include homodyne interferometry and heterodyne interferometry. The key difference between them lies in whether the reference light is loaded on a carrier. In heterodyne interferometry, the reference light is loaded on a carrier, which endows it with strong anti-interference ability and high sensitivity. Therefore, heterodyne interferometry is often adopted in measurement optical paths, and its composition principle is shown in Figure 1.

The monochromatic light emitted by the laser source (Laser, 633 nm) is split into reference light (green line) and measurement light (red line) by the polarizing beam splitter (PBS 1). The reference light undergoes frequency modulation via an acousto-optic frequency shifter, with a frequency shift of $f_{0}$, and then directly enters the photodetector. The measurement light passes through the polarizing beam splitter (PBS 2), a quarter-wave plate, and a mirror (Mirror 2), traversing the refractive index measurement region. It is then vertically incident on a mirror (Mirror 3), reflects, and returns along the same path to the polarizing beam splitter (PBS 2). The measurement light interferes with the reference light at the beam splitter (BS 1), and the interference signal carries information about the refractive index variation in the measurement region.

The expression of the reference light at the photodetector is as follows:

(1) $$E_{r}\left(t\right)=E_{r0}cos\left[2\pi \left(\frac{c}{\lambda }+f_{0}\right)t+\varphi_{0}+\varphi_{1}\right]$$

The expression of the measurement light at the photodetector is as follows:

(2) $$E_{m}\left(t\right)=E_{m0}cos\left[2\pi \left(\frac{c}{\lambda }\right)t+\varphi_{0}+\varphi_{2}+\frac{2\pi n\left(t\right)\cdot 2D}{\lambda }\right]$$

Assuming the internal refractive index of the system is 1, in Equations (1) and (2), $E_{r0}$ and $E_{m0}$ represent the electric field intensity amplitudes corresponding to the reference light and measurement light, respectively; c denotes the speed of light; λ represents the laser wavelength; $f_{0}$ is the frequency shift introduced by the acousto-optic frequency shifter; $\varphi_{0}$ is the initial phase of the laser; $\varphi_{1}$ is the phase change caused by the propagation path of the reference light within the system environment; $\varphi_{2}$ is the phase change caused by the propagation path of the measurement light within the system environment; $n\left(t\right)$ denotes the refractive index variation in the probe region; and D represents the length of the probe region.

After interference between the reference light and measurement light at the beam splitter (BS 1), the photodetector converts the interference signal into a photoelectric current as follows:

(3) $$i\left(t\right)={\alpha \left(E_{r}\left(t\right)+E_{m}\left(t\right)\right)}^{2}=\alpha \left[\frac{E_{r0}^{2}}{2}+\frac{E_{m0}^{2}}{2}+E_{r0}E_{m0}\cos\left(2\pi f_{0}t+\varphi_{1}-\varphi_{2}-\frac{2\pi n\left(t\right)\cdot 2D}{\lambda }\right)\right]$$

α is the photoelectric conversion coefficient of the photodetector.

### 2.2. Signal Processing of Sea Water Refractive Index Based on SDR

**Figure 2:**
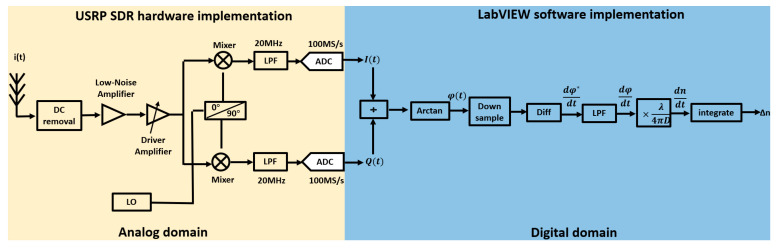
Demodulation algorithm flow based on SDR.

This paper employs the Universal Software Radio Peripheral Software-Defined Radio (USRP-SDR) as the hardware platform for refractive index signal demodulation and utilizes NI LabVIEW 2019 (64-bit) as the software platform. The specific demodulation algorithm flowchart is shown in Figure 2.

The photoelectric current signal $i\left(t\right)$ is DC-filtered, as follows:

(4) $$i_{A}\left(t\right)=\alpha \left[E_{r0}E_{m0}\cos\left(2\pi f_{0}t+\varphi_{1}-\varphi_{2}-\frac{2\pi n\left(t\right)\cdot 2D}{\lambda }\right)\right]$$

The AC signal $i_{A}\left(t\right)$ is then amplified by a radio frequency (RF) amplifier and split into two paths. Each path is mixed with the sine and cosine signals generated by the local oscillator (LO) at frequency $f_{L}$, specifically $sin2\pi f_{L}t$ and $cos2\pi f_{L}t$, respectively, to perform the down-conversion. After mixing, the high-frequency components resulting from the mixing process are removed using a low-pass filter (LPF), yielding the signals $I\left(t\right)$ and $Q\left(t\right)$ as follows:

(5) $$I\left(t\right)=i_{A}\left(t\right)cos\left(2\pi f_{L}t\right)=\alpha E_{r0}E_{m0}\cos({2\pi \left(f_{L}-f_{0}\right)t+\varphi }_{2}-\varphi_{1}+\frac{2\pi n\left(t\right)\cdot 2D}{\lambda })$$

(6) $$Q\left(t\right)=i_{A}\left(t\right)sin\left(2\pi f_{L}t\right)=\alpha E_{r0}E_{m0}\sin(2\pi \left(f_{L}-f_{0}\right)t+\varphi_{2}-\varphi_{1}+\frac{2\pi n(t)\cdot 2D}{\lambda })$$

The low–intermediate frequency (LIF) receiver refers to a device that processes signals in the low–intermediate frequency band, while a zero–intermediate frequency (ZIF) receiver refers to one that processes signals at the baseband. Low–intermediate frequency receivers effectively address local oscillator leakage and flicker noise issues present in zero–intermediate frequency receivers, thereby improving the signal-to-noise ratio (SNR). Therefore, the demodulation process employs a low–intermediate frequency structure, where $f_{L}\neq f_{0}$.

**Figure 3:**
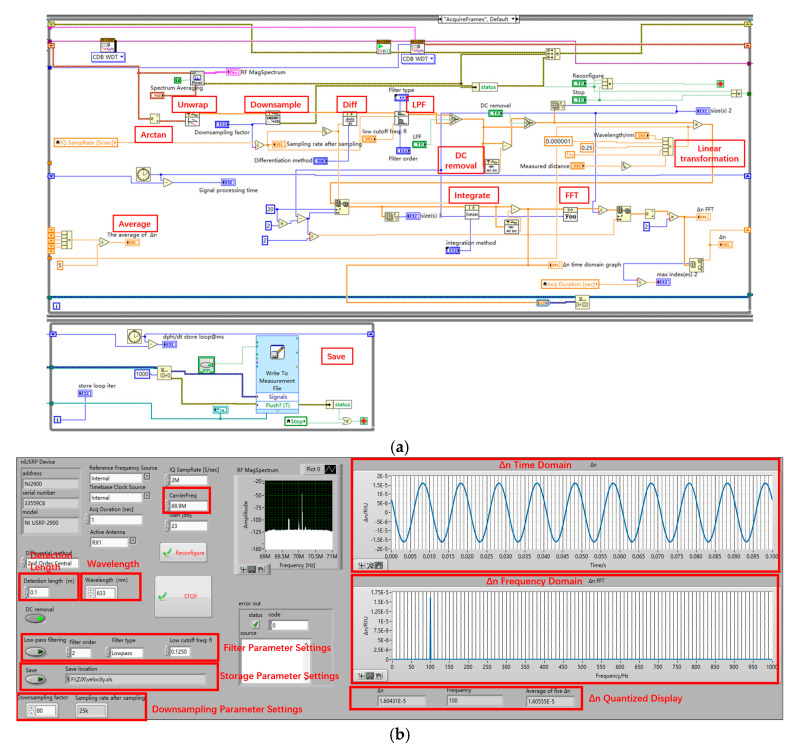
Signal processing flow based on LabVIEW. (**a**) LabVIEW block panel; (**b**) LabVIEW front panel.

The analog signals $I\left(t\right)$ and $Q\left(t\right)$ are converted to digital signals using an analog-to-digital converter (ADC). These digital signals are then processed on the upper computer using LabVIEW. The program implementation is as shown in Figure 3.

In the software, $I\left(t\right)$ and $Q\left(t\right)$ are divided, and an arctangent calculation is performed to extract the phase information from the AC signal. However, due to the range characteristics of the arctangent function, the phase obtained directly will experience wrapping. To address this, the unwrap module in LabVIEW is used to obtain the phase as follows:

(7) $$\varphi \left(t\right)=2\pi \left(f_{L}-f_{0}\right)t+\frac{2\pi n\left(t\right)\cdot 2D}{\lambda }+\Psi$$

Ψ is the initial phase value, where $\Psi =\varphi_{2}-\varphi_{1}$. The frequency of $\varphi \left(t\right)$ is determined by n(t). The refractive index of seawater in the ocean is closely related to temperature, salinity, and other factors. Since temperature and salinity changes in the ocean are slow processes, the variation signal of the seawater refractive index is a low-frequency signal, and the frequency of n(t) is within 100 Hz. The minimum sampling rate of USRP SDR is 200 KS/sec; an excessively high sampling rate will degrade computational performance and introduce noise. Therefore, after obtaining the phase $\varphi \left(t\right)$, an appropriate decimation factor is set to adjust the sampling rate.

The value of Ψ is not only related to the optical propagation paths of the reference light and measurement light within the system environment but also influenced by noise and the unwrapping algorithm. As a result, we are unable to obtain the accurate initial phase value. The actual refractive index measurement results are typically characterized by the refractive index change rate $\frac{dn}{dt}$. By differentiating Equation (7), we can obtain an implicit expression for the refractive index change rate as follows:

(8) $$\frac{d\varphi^{*}}{dt}=2\pi \left(f_{L}-f_{0}\right)+\frac{4\pi D}{\lambda }\cdot \frac{dn\left(t\right)}{dt}$$

Differentiation operations on discrete points amplify the high-frequency components of the signal while leaving the low-frequency components unchanged. Therefore, a low-pass filter (LPF) is added after differentiation to remove the high-frequency signals.

Observing Equation (8), it can be seen that the phase change rate contains not only refractive index information but also a DC offset of $2\pi \left(f_{L}-f_{0}\right)$. This DC offset is removed using the DC removal module in LabVIEW to obtain the following:

(9) $$\frac{dn}{dt}=\frac{\lambda }{4\pi D}\cdot \frac{d\varphi }{dt}$$

In this case, $\frac{d\varphi }{dt}=\frac{d\varphi^{*}}{dt}-2\pi \left(f_{L}-f_{0}\right)$.

After integration, the change in refractive index is obtained as follows:

(10) $$∆n=\int \frac{dn\left(t\right)}{dt}dt=\int \frac{\lambda }{4\pi D}\cdot \frac{d\varphi }{dt}dt$$

## 3. Simulation of SDR-Based Demodulation Algorithm for Seawater Refractive Index Variations

Simulations of the orthogonal demodulation algorithm were conducted on the MATLAB R2021a platform. Based on Equation (9), the variation rate of the seawater refractive index $\frac{dn}{dt}$ was assumed to be a cosine function as follows:

(11) $$\frac{dn}{dt}=\frac{\lambda }{4\pi D}\cdot 2\pi A_{m}\cos\left(2\pi f_{m}t\right)=\frac{\lambda }{2D}\cdot A_{m}\cos\left(2\pi f_{m}t\right)$$

where $A_{m}$ is the frequency deviation amplitude.

The photoelectric current signal modulated by seawater refractive index is obtained.

(12) $$i_{A}\left(t\right)=Acos\left(2\pi f_{0}t+\frac{4\pi D}{\lambda }\int \frac{dn}{dt}dt\right)=Acos\left(2\pi f_{0}t+\frac{A_{m}}{f_{m}}\sin\left(2\pi f_{m}t\right)\right)$$

A is the amplitude of the carrier signal, $f_{0}$ is the frequency of the carrier signal, and $\frac{A_{m}}{f_{m}}$ is the amplitude of the phase variation. In the actual system, the carrier power is −20 dBm, corresponding to A=0.0224, and the carrier frequency $f_{0}=70$ MHz. Therefore,

(13) $$i_{A}\left(t\right)=0.0224\times cos\left(2\pi \times 70\times 10^{6}t+B\sin\left(2\pi f_{m}t\right)\right)$$

**Figure 4:**
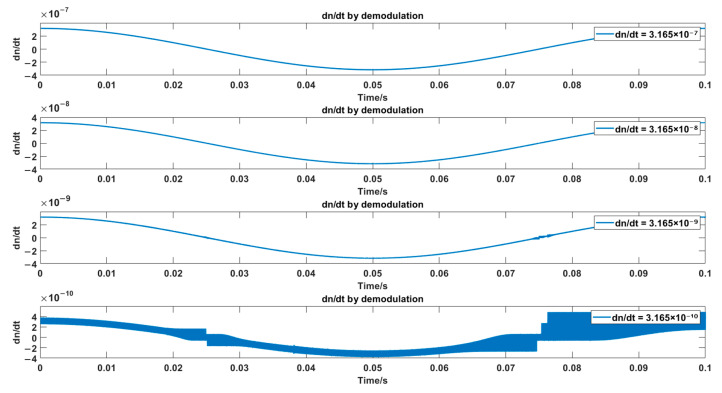
Simulation results of different dn/dt values using IQ Arctan demodulation.

During the simulation process, the low-pass filter is a Butterworth filter with an order of 12, a cut-off frequency of 1 kHz, and a passband ripple of 0.2 dB. The local oscillator frequency $f_{L}=69.9$ MHz, $f_{m}=10$ Hz, and the detection range D is set to 0.1 m. To obtain the minimum refractive index variation rate based on IQ demodulation, different refractive index variation rates are set with a step size of one order of magnitude. The demodulated refractive index variation rate signals are compared with the set refractive index variation rate signals, and the results are shown in Figure 4.

It can be observed that at dn/dt = $3.165\times 10^{-10}$, the signal shows significant distortion. It can be concluded that the resolution of the refractive index variation rate based on IQ arctan demodulation is $3.165\times 10^{-9}$ RIU/s.

**Figure 5:**
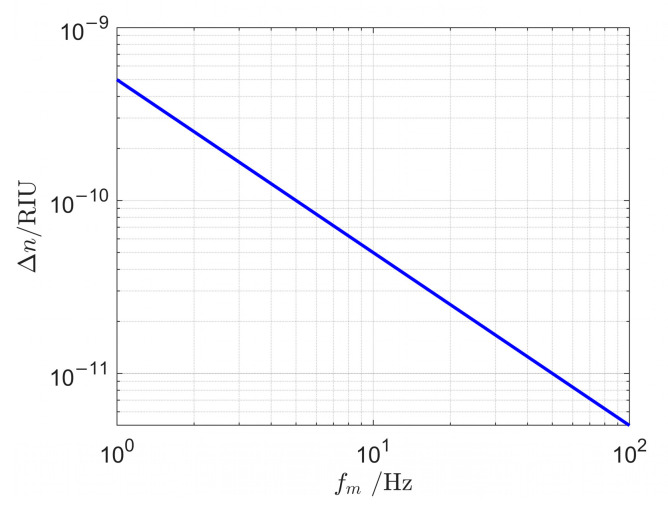
Seawater refractive index variation rate demodulation limit at different frequencies.

Based on Equation (10), the demodulated refractive index variation limits at different frequencies are calculated, as shown in Figure 5.

Thus, within the frequency range of 1 Hz to 100 Hz, the resolution of refractive index variation in seawater achieves $5\times 10^{-10}$ RIU.

## 4. Calibration of Optical Phase and Refractive Index Variations for Seawater Refractive Index Measurement Using SDR

A signal generator (Tek4102A RF signal generator, Beaverton, OR, USA) is used to generate a sine RF signal to simulate the optoelectronic current frequency modulation (FM) signal. The carrier frequency is set to 70 MHz, and the power is −20 dBm. The optical phase expression is as follows:

(14) $$\varphi \left(t\right)=2\pi f_{0}t+\frac{A_{m}}{f_{m}}\sin\left(2\pi f_{m}t\right)$$

The frequency deviation $A_{m}$ is set according to Formula (11), and the modulation frequency $f_{m}$ ranges from 1 Hz to 100 Hz.

The IQ sampling rate is 2 MS/s, with a downsampling factor set to 80. D set to 0.1 m. The refractive index variation is calculated using Formula (10), as follows:

(15) $$∆n=\int \frac{\lambda }{4\pi D}\cdot \frac{d\varphi }{dt}dt=\frac{\lambda }{4\pi D}\cdot \frac{A_{m}}{f_{m}}\sin\left(2\pi f_{m}t\right)$$

French scholars’ research on the ocean indicates that the precision of seawater refractive index measurements should be at least $6\times 10^{-7}$ RIU to cover the entire ocean range [22], thus setting ∆n = $10^{-5}$, $10^{-6}$, $10^{-7}$, and $10^{-8}$ RIU levels.

**Figure 6:**
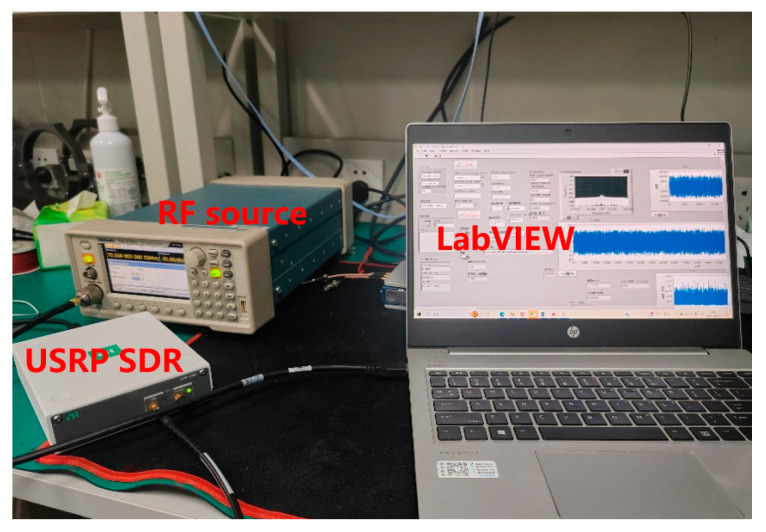
Scenario of verifying the demodulation algorithm with a radio frequency source.

The experimental setup is depicted in Figure 6.

**Figure 7:**
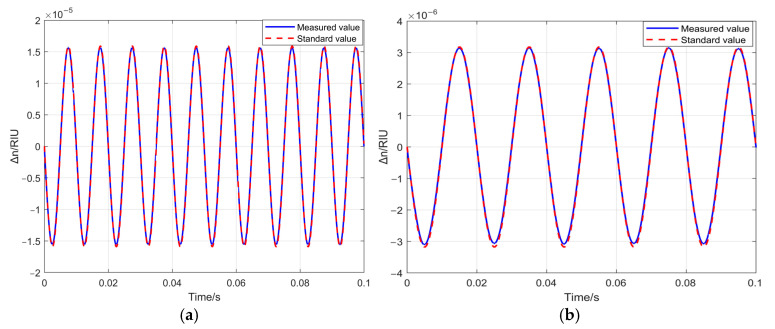

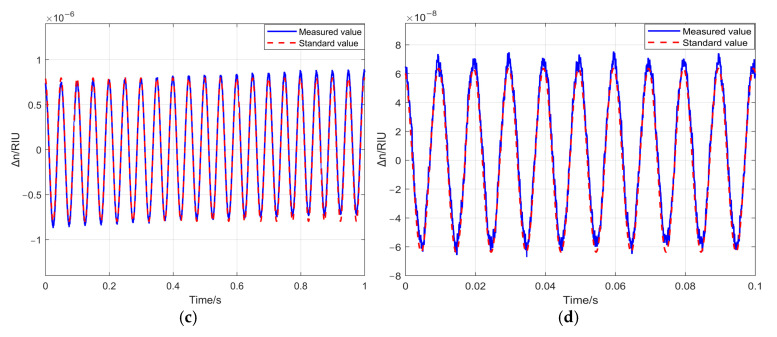
Standard values and SDR measurement values corresponding to typical refractive index changes. (**a**) Comparison between the waveform measured by SDR and the standard waveform when $∆n={1.59e}^{-5}$ RIU; (**b**) comparison between the waveform measured by SDR and the standard waveform when $∆n={3.18e}^{-6}$ RIU; (**c**) comparison between the waveform measured by SDR and the standard waveform when $∆n={1.59e}^{-7}$ RIU; (**d**) comparison between the waveform measured by SDR and the standard waveform when $∆n={6.37e}^{-8}$ RIU.

By adjusting the frequency deviation $A_{m}$ and the modulation frequency $f_{m}$, different refractive index variation signals are generated. Several typical refractive index variations are selected, with the corresponding $A_{m}$ and $f_{m}$ values as shown in the Table 1:

The demodulated waveform is shown in Figure 7.

As ∆n decreases, the overlap between the standard value and the measured value from SDR demodulation reduces. This is because the spectral purity of the signal generator declines, and the noise influence becomes more prominent as ∆n decreases. By adjusting the frequency deviation and rate, multiple specific values are established under the same refractive index variation. The relative error E of the demodulation results is defined as follows:

(16) $$E=\frac{\left|{Peak}_{measure}-{Peak}_{standard}\right|}{{Peak}_{standard}}\times 100\%$$

**Figure 8:**
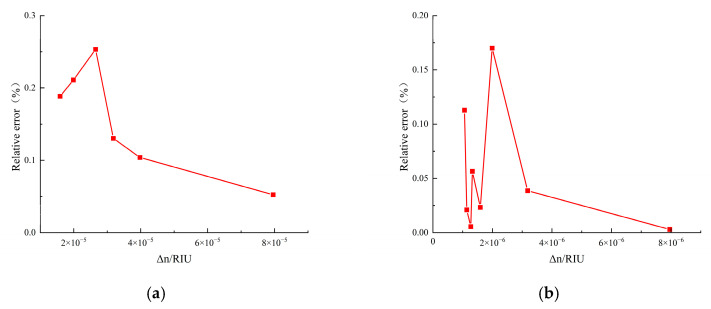

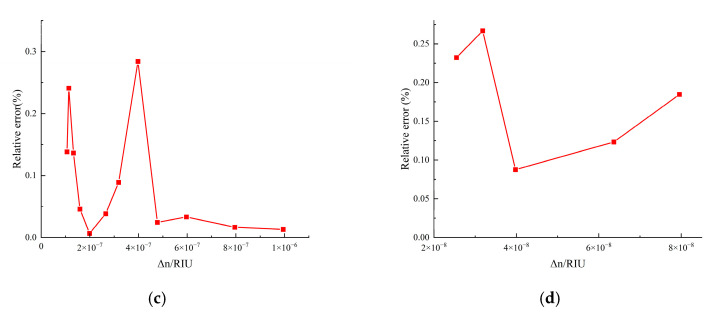
Results of verifying the demodulation algorithm with a radio frequency source. (**a**) The relative error of demodulation when $∆n=10^{-5}$ level; (**b**) the relative error of demodulation when $∆n=10^{-6}$ level; (**c**) the relative error of demodulation when $∆n=10^{-7}$ level; (**d**) the relative error of demodulation when $∆n=10^{-8}$ level.

To eliminate the influence of noise, the ${Peak}_{measure}$ is calculated by averaging the peak-to-peak values obtained from five measurements and then dividing the result by 2. ${Peak}_{standard}$ is the set standard peak value. The results are shown in Figure 8.

It can be found that under different levels, the relative errors of the amplitudes of the demodulation results are all within 0.3%. It is found that under different refractive index variations, the relative errors of demodulation result amplitudes are within 0.3%. This error is due to two main factors: one is the noises from the RF source itself (e.g., spurious, thermal, and phase noises), causing fluctuations between the generated RF and the set value; the other is the electromagnetic interference from ambient noise during demodulation and the noise in channels generated during SDR software processing (e.g., filtering and down-sampling). As most of these are low-frequency noises, a filter can be designed later to reduce their impact on measurements.1

## 5. Experimental System for Seawater Refractive Index Measurement Based on SDR

**Figure 9:**
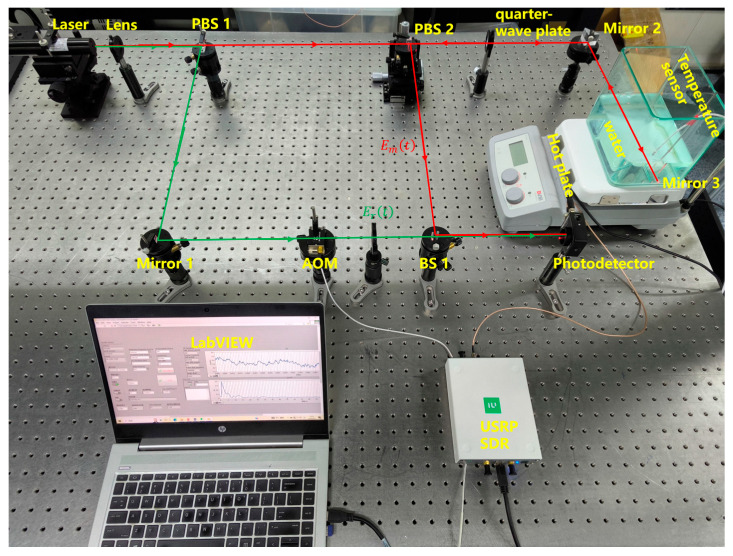
Test system. The measurement beam is represented by red arrows, and this beam pass through the seawater measurement area. The reference beam is represented by green arrows.

Based on Figure 1, an optical coherent measurement system for seawater refractive index was constructed using software-defined radio (SDR) as shown in Figure 9. The detection range was 0.17 m. Deionized water’s high purity allows for precise control of experimental conditions. Moreover, both deionized water and seawater are transparent media and follow the same principle in the physical process of laser interference. Hence, deionized water can be used as the test target. Reflective tape was attached to the container glass to act as Mirror 3. Temperature adjustments were made using a heating platform to induce changes in the refractive index, and a temperature sensor was used to measure the real-time temperature of the deionized water. After entering the photodetector, the interference signals were processed sequentially through the USRP-SDR and LabVIEW for demodulation, ultimately yielding information on the refractive index variations.

During the experiment, the temperature sensor used was the MOBO Robotics, mSTS-P model, with a measurement accuracy of 0.001 °C, a sampling rate of 1 Hz, and a sampling duration of 35 s. The temperature varied from 24.460 °C to 24.950 °C. The traditional empirical formula for fitting the refractive index of water with temperature, as referenced in formula (17), was employed for the analysis [23].

(17) nT,λ=1.76163162−0.011988λ2+0.00644277λ2−0.014911912−6.3649−10.562×λ−0.589263λ−0.1221145T−203T+65.7081×107−2352.12−143.63×λ−0.58926×1+0.4436λ−0.1221145T−202T+65.7081×107−76087.9−12504×λ−0.58926×1+0.08430λ−0.1221145T−20T+65.7081×107

**Figure 10:**
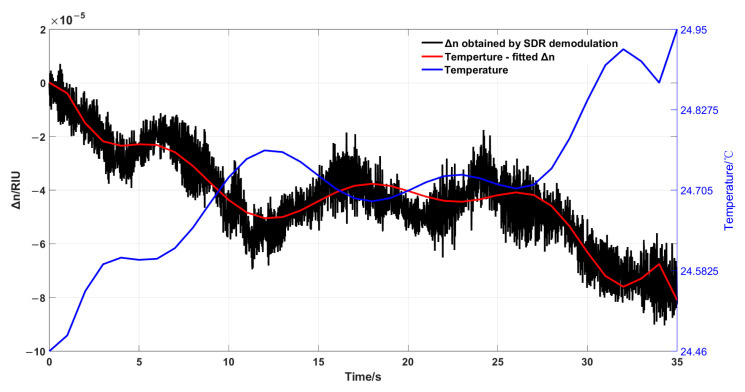
Curves of refractive index change fitted by temperature and refractive index change obtained by SDR demodulation.

T represents the temperature of the deionized water, and λ represents the laser wavelength, which is 633 nm. The refractive index variation is calculated as ∆n = $10^{-5}$ RIU under the given temperature changes. The curves representing the refractive index variation derived from temperature fitting and the refractive index variation obtained through SDR demodulation are illustrated in Figure 10.

As shown in the figure, the blue curve representing temperature is strongly correlated with the red curve representing refractive index. Furthermore, the results obtained from SDR demodulation are consistent with those derived from temperature fitting. Due to the higher sampling frequency of the SDR system (on the order of kHz), the refractive index variations obtained from SDR demodulation reveal more detailed information compared to those from temperature fitting. Taking the refractive index variation obtained by temperature fitting as the standard value and the down-sampled refractive index variation obtained by SDR demodulation as the measured value, the root mean square error (RMSE) can reflect the average level of the deviation between the measured values and the standard values, and the mean square error between the two is calculated as follows:

(18) $$RMSE=\sqrt{\frac{1}{n}\sum_{i=1}^{n}{\left(y_{p}-\hat{y_{s}}\right)}^{2}}$$

n represents the number of sampling points, $y_{p}$ represents the refractive index variations obtained from temperature fitting, and $\hat{y_{s}}$ represents the refractive index variations derived from SDR demodulation. The calculated RMSE for the refractive index variations obtained from SDR demodulation is ${5.68\times 10}^{-6}$ RIU. This means that the average error between the refractive index variation obtained through SDR demodulation and that obtained by fitting with temperature is approximately ${5.68\times 10}^{-6}$ RIU. The experiment demonstrates the effectiveness of using SDR for refractive index demodulation. However, the actual refractive index of seawater is affected not only by temperature but also by salinity (S) and pressure (P). Thus, $n\left(T,\lambda \right)$ in Equation (17) becomes $n\left(T,\lambda ,S,P\right)$. In subsequent experiments, seawater with different salinities will be simulated by adding salts at different concentrations to deionized water. A temperature-control device will be used to regulate the seawater temperature, and a pressure chamber will be employed to simulate the seawater pressure at different depths, thereby generating realistic seawater conditions.

## 6. Conclusions

This paper employs software-defined radio (SDR) demodulation for high-precision measurements of changes in seawater refractive index. Using USRP-SDR as the hardware demodulation platform and LabVIEW as the software demodulation platform, the system achieves high-precision measurements for four orders of magnitude: ∆n = $10^{-5}$ RIU, $10^{-6}$ RIU, $10^{-7}$ RIU, and $10^{-8}$ RIU. By simulating seawater refractive index signals with an RF signal source, the results demonstrate that the relative error of the SDR demodulation results is within 0.3% for the specified refractive index variations. Using heated, deionized water as the test target, the experiment validates that the root mean square error (RMSE) of the entire system, from optics to electronics, is $5.68\times 10^{-6}$ RIU, thereby realizing high-precision measurements of seawater refractive index variations. This study provides a novel demodulation method for achieving high-precision measurements of seawater refractive index variations under different environmental conditions. It helps researchers better understand seawater’s physical properties and dynamic changes, offering key data for ocean circulation model construction and marine ecological environment monitoring. It also aids in studying ocean–atmosphere interactions and improving climate prediction accuracy.

However, this study has certain limitations. From a system perspective, the optical path is not yet integrated, and the current measurement system has poor portability, restricting its engineering applications. Additionally, during the measurement of a real seawater refractive index, seawater contains various impurities and suspended solids such as sediment, algae, and microorganisms. These impurities and suspended solids scatter and absorb light, affecting light propagation and refraction in seawater, thus increasing the uncertainty of measurement results. This issue also needs to be considered in future work.

## Figures and Tables

**Table 1:** Typical values of Δn and the corresponding $A_{m}$.

∆n/RIU	$A_{m}/kHz$	$f_{m}/Hz$
${1.59e}^{-5}$	3.15956	100
${3.18e}^{-6}$	0.31956	50
${1.59e}^{-7}$	0.00631	20
${6.37e}^{-8}$	0.01264	100

## Data Availability

The original contributions presented in this study are included in the article.
